# Insights into the relationship between emotional intelligence and critical thinking among nursing students

**DOI:** 10.1186/s12912-025-03782-7

**Published:** 2025-08-23

**Authors:** Ahmad Ayed, Ibrahim Aqtam, Malakeh Z. Malak, Dalia Toqan, Bahaaeddin M. Hammad, Jamal Qaddumi, Mustafa Shouli

**Affiliations:** 1 Faculty of Nursing, Arab American University, Jenin, Palestine; 2 Ibn Sina College for Health Professions, Department of Nursing, Nablus University for Vocational and Technical Education, Nablus, Palestine; 3 Community Health Nursing, Faculty of Nursing, Al-Zaytoonah University of Jordan, Amman, Jordan; 4 Faculty of Medicine and Health Sciences, An-Najah National University, Nablus, Palestine

**Keywords:** Emotional intelligence, Critical thinking, Nursing education, Resource-constrained settings, Clinical decision-making

## Abstract

**Background:**

Emotional intelligence (EI) and critical thinking (CT) are critical skills for nursing students, but the specific mechanisms underlying their interaction remain unclear in resource-constrained settings like Palestine, where unique contextual factors may influence their relationship.

**Objective:**

This study examined the relationship between EI and CT disposition among nursing students at the Arab American University, Palestine.

**Methods:**

A cross-sectional design was utilized, with 288 s- to fourth-year nursing students (a 96% response rate) being recruited. Validated scales, the Schutte Self-Reported Emotional Intelligence Test (SSEIT) and Critical Thinking Disposition Scale (CTDS), were utilized. Data were analyzed using Pearson’s correlation and multiple linear regression analysis (F = 89.47, *p* < 0.001).

**Results:**

Participants exhibited high EI (M = 122.5 ± 16.1) and moderate CT (M = 43.1 ± 6.1). EI and CT were strongly positively correlated (*r* = 0.683, *p* < 0.001). Regression analysis identified EI (β = 0.658, *p* < 0.001), being male (β = 0.131, *p* = 0.002), and having a family member working in healthcare (β = 0.087, *p* = 0.043) as significant predictors of CT disposition.

**Conclusion:**

EI demonstrates a strong correlation with CT among Palestinian nursing students. Integrating EI training into curricula is associated with higher CT disposition and may be related to differences in clinical decision-making in high-pressure healthcare settings.

**Clinical trial number:**

Not applicable.

## Introduction

Emotional intelligence (EI) and critical thinking (CT) are essential competencies for nursing students, enabling clinical decision-making, patient communication, and resilience in high-stress environments [1, 2]. EI encompasses perceiving, regulating, and utilizing emotions, while CT involves systematic analysis, logical reasoning, and reflective judgment [3, 4]. Both skills are critical in nursing practice, where rapid decision-making under emotional and resource constraints directly impacts patient outcomes [5, 6]. Specifically, the theoretical framework suggests that EI facilitates CT through enhanced emotional regulation, which reduces cognitive load during stressful clinical situations, thereby enabling nurses to engage in more systematic analytical reasoning [7]. Benner et al. emphasize that nursing practice requires the integration of emotional competence with analytical thinking, particularly in complex clinical situations where emotional responses can either enhance or impair clinical reasoning [2]. This integration becomes especially crucial in high-pressure environments where nurses must simultaneously manage their emotional responses while making critical clinical decisions. For instance, nurses with higher EI demonstrate enhanced clinical decision-making in high-stakes settings such as neonatal intensive care units and emergency departments, underscoring EI’s role in mitigating cognitive overload during crises [6, 8].

The synergistic relationship between EI and CT operates through several specific mechanisms. First, emotional regulation, a core component of EI, enables nurses to maintain cognitive clarity during high-stress situations, facilitating systematic problem-solving and analytical reasoning [6]. Second, the self-awareness aspect of EI promotes metacognitive reflection, which is fundamental to critical thinking processes [9]. Third, social awareness components of EI enhance perspective-taking abilities, which support the intellectual empathy required for comprehensive clinical reasoning [10]. Research demonstrates that stronger EI is associated with more successful problem-solving, reduced cognitive error, and greater adaptability in nursing practice [10, 11]. EI, for example, fosters emotional regulation during crises, which allows nurses to engage in analytical reasoning [6]. Conversely, CT disposition may enhance EI through the facilitation of self-reflection and empathy [9]. However, several specific aspects of the EI-CT relationship remain understudied, particularly: (1) how different components of EI (emotion perception, regulation, utilization) differentially contribute to various CT dimensions, (2) the role of contextual stressors in moderating this relationship, and (3) whether cultural factors in non-Western settings influence the mechanisms through which EI supports CT development. Despite this interdependence, there has been little empirical work examining the specific mechanisms underlying EI’s influence on CT development, particularly in resource-poor contexts [12, 13].

The significance of this study lies in its potential to inform evidence-based curriculum development for nursing education in resource-constrained environments. Understanding the EI-CT relationship in the Palestinian context can guide the development of targeted interventions that leverage emotional competencies to enhance analytical thinking skills, ultimately improving clinical preparedness among nursing graduates who will work in challenging healthcare environments.

Palestinian nursing education faces unique challenges, which include political instability, overcrowded hospitals, and traditional pedagogical approaches emphasizing rote learning [14, 15]. These systemic stressors, including frequent interruptions due to political unrest, create high-stress learning environments where emotional resilience becomes a critical competency rather than merely an educational goal. Students often experience limited opportunities for reflective practice or emotional skill development, which may hinder CT development [16]. Specifically, the limited clinical training opportunities due to overcrowded hospitals and restricted access to clinical sites during periods of political unrest may create a unique educational context where family healthcare exposure becomes particularly influential in developing clinical reasoning skills. The presence of stressors like pediatric clinical training anxiety and inadequate coping skills exacerbates these challenges, necessitating targeted interventions [8, 17]. Moreover, exposure to healthcare professions within families and cultural norms may differently affect students’ emotional and cognitive competencies compared to those in global settings [18]. Understanding how EI and CT correlate in this context is crucial for designing curricula that are contextually relevant and for enhancing clinical preparedness within resource constraints.

This study addresses these knowledge gaps by examining the relationship between EI and CT disposition among nursing students at the Arab American University, Palestine. By investigating how EI and sociodemographic factors (e.g., gender, healthcare exposure in the family) predict CT, the findings aim to inform evidence-based integration of EI training in nursing education to enhance clinical decision-making in resource-scarce settings.

## Methods

### Design and setting

A cross-sectional study was conducted at the Arab American University between March 23 and April 9, 2025. This timeframe was selected to coincide with the mid-semester period when students had sufficient clinical exposure while avoiding examination periods that might affect participation rates. Arab American University is the largest nursing school in Palestine, with a student body of 900 nursing students. The university offers a four-year nursing program that culminates in a baccalaureate degree in nursing, with the first year dedicated to general requirements.

### Population and sampling

The initial sample size for this study was determined using Raosoft software, with a population size of 900 and a response distribution of 50%. With a margin of error of 5% and a confidence interval of 95% [19], the estimated sample size was calculated to be 270 participants. To account for potential attrition, a convenience sample of 300 students was enrolled. Convenience sampling was chosen due to practical constraints in accessing nursing students across different academic years and the need to ensure voluntary participation without disrupting academic schedules. While convenience sampling limits generalizability, several factors support the sample’s representativeness: (1) proportional recruitment across second, third, and fourth-year students ensured representation of different academic levels, (2) the high response rate (96%) minimized selection bias, and (3) demographic characteristics of participants were consistent with the broader nursing student population at the university. However, we acknowledge that non-responders may have differed systematically from participants, potentially affecting the generalizability of findings. Future studies should employ probability sampling methods and conduct non-responder analyses to enhance external validity. Additionally, the single-institution design limits generalizability to other Palestinian universities or international contexts, necessitating multi-site replication studies. However, despite these precautions, 288 students completed the study and returned the questionnaires. The sample was proportionally gathered from second, third, and fourth-year nursing students, ensuring representation across different academic levels within the nursing program.

### Inclusion and exclusion criteria

#### Inclusion criteria

Participants in this study were nursing students enrolled at the Arab American University, specifically in the second, third, or fourth year of their nursing program. Students were required to have completed at least one clinical rotation, ensuring they had sufficient clinical exposure to demonstrate the clinical reasoning skills that CT measures aim to assess. Participants also needed to be willing to provide informed consent and participate voluntarily in the study. Only students who were actively enrolled in the nursing program during the study period were included.

#### Exclusion criteria

Nursing students who were in their first year, as they were still completing general requirements and had not yet engaged in clinical rotations, were excluded from the study. This exclusion was necessary because clinical exposure is theoretically linked to the development of both EI and CT disposition in nursing practice, and students without clinical experience may not have had adequate opportunities to develop or demonstrate these competencies in healthcare contexts. Our findings support this rationale, as participants with clinical experience demonstrated measurable EI and CT competencies. Clinical training provides real-world scenarios where students must regulate emotions under pressure (developing EI) while simultaneously engaging in analytical reasoning for patient care decisions (developing CT). The moderate CT scores observed in our study likely reflect varying levels of clinical exposure across academic years, with more experienced students having greater opportunities to integrate emotional regulation with systematic problem-solving in actual healthcare settings. Additionally, students who were not currently enrolled in the nursing program or had taken a leave of absence were excluded.

#### Instruments

The instrument consisted of three components, with the first containing demographic data such as gender and age. The Schutte Self-Reported Emotional Intelligence Test (SSEIT) constituted the second component. Schutte et al. developed the SSEIT to assess emotional intelligence (EI) [20]. It has 33 items distributed across four subscales: “perception of emotions, social skills, self-management of emotions, and emotion utilization.” Each item has a five-point Likert-type rating system. The overall scale scores ranged from 33 to 165. To calculate the final score, values for items 5, 28, and 33 must be reversed. The scores range from 33 to 165, and higher scores indicate higher levels of EI. With a Cronbach’s alpha of 0.90, the instrument is considered valid and reliable [20–22].

The third component included the Critical Thinking Disposition Scale (CTDS). This instrument consisted of 11 items designed to measure the CT disposition of students enrolled in nursing courses [23]. The CTDS encompasses Critical openness (seven items) and Reflective skepticism (four items). Scores were determined using a Likert scale with the following ranges: 1 = strongly disagree, 2 = disagree, 3 = neutral, 4 = agree, and 5 = strongly agree. The total score for the Critical Openness scale ranges from 7 to 35 with the following cut-offs (7–21 low; 22–28 moderate; 29–35 high). Reflective Skepticism ranges from 4 to 20 with cut-off ranges being 4–12 low; 13–16 moderate; and 17–20 high. The scale is valid and reliable [12, 23, 24].

A pilot study with 30 students was conducted to ensure comprehension and cultural appropriateness of the scales in the Palestinian context. No significant comprehension issues were identified during the pilot phase. In the current study, the Cronbach alpha for CTDS was 0.86 and 0.88 for SSEIT.

### Data collection procedure

Once approval was obtained to conduct the study, nursing students were recruited to participate. To address potential concerns about perceived coercion, the recruitment process was carefully designed to ensure voluntary participation under strict ethics committee oversight. While initial contact was facilitated through the vice dean’s office for administrative purposes, the researcher personally met with students in their classrooms to explain the study’s purpose and emphasize that participation was entirely voluntary with no impact on their academic standing. The university’s ethics committee monitored the recruitment process to ensure no coercive practices occurred, and students were provided with independent contact information for the ethics committee should they have concerns about the recruitment process. Students were given adequate time to consider participation, and those who expressed interest were provided with detailed information sheets. Subsequently, the purpose of the study was thoroughly discussed with the nursing students. The researcher then administered paper-based questionnaires in English, as all participants studied in English.

### Ethical considerations

This study adhered to the principles of the Helsinki Declaration. The research process involved obtaining all necessary ethical approvals, including permission from the instrument developers to use their validated tools, Institutional Review Board (IRB) approval from the Arab American University (Reference Number: J-2025/A/8/N), and approval from relevant university authorities. Written informed consent was obtained from all participants after they were fully informed about the study’s purpose, procedures, potential benefits, and possible risks. Participation was entirely voluntary, and participants had the right to withdraw from the study at any time without penalty. To ensure confidentiality and anonymity, each questionnaire was assigned a code number, and all collected data were securely stored and used solely for research purposes.

### Data analysis

Data were analyzed using SPSS v.26. Descriptive statistics (mean, SD, frequencies) summarized demographic and scale scores. Pearson’s correlation assessed relationships between variables. Multiple linear regression identified predictors of critical thinking (dependent variable). Assumptions of normality, multicollinearity, and homoscedasticity were met (no missing data/outliers).

## Results

### Participants’ characteristics

Out of 300 nursing students invited to participate, 288 successfully completed the study, resulting in a high response rate of 96.0%. The participants had a mean age of 22.7 ± 1.8 years. The sample included 150 females (52.1%). Regarding the academic level, 108 students (37.5%) were in their fourth year. Additionally, 197 participants (68.4%) reported having a family member working in the medical or nursing field. The majority of students, 259 (89.9%), expressed an interest in nursing, as outlined in Table 1.

**Table 1:** Demographic characteristics of the participants (*N* = 288)

Characteristics		*N* (%)	M(SD)
Age			22.7(1.8)
Gender	Male	138 (47.9)	
	Female	150 (52.1)	
Academic year	Second year	105 (36.5)	
	Third year	75(26.0)	
	Fourth year	108 (37.5)	
Medical or Nursing Personnel in the Family	Yes	197 (68.4)	
	No	91 (31.6)	
Interest in nursing	Yes	259 (89.9)	
	No	29 (10.1)	

The analysis revealed that the mean emotional intelligence score was 122.5 ± 16.1, ranging from 33 to 165, indicating a high level of emotional intelligence among participants. Among its domains, “Utilizing emotions” had the highest mean score (51.4 ± 7.0), while “Perception of emotions” had the lowest (19.6 ± 3.0). Regarding critical thinking, the mean score was 43.1 ± 6.1, reflecting a moderate level of critical thinking ability. Within its subcomponents, “Critical openness” was at a moderate level (27.4 ± 4.2), and “Reflective skepticism” also showed a moderate level (15.7 ± 2.4), as presented in Table 2.

**Table 2:** Distribution of emotional intelligence and critical thinking (*N* = 288)

Variable	M	SD
**Total Emotional Intelligence**	122.5	16.1
Perception of emotions	19.6	3.0
Social skills or managing others’ emotions	25.7	4.3
Managing emotions in the self	25.8	5.7
Utilizing emotions	51.4	7.0
**Total Critical Thinking**	43.1	6.1
Critical Openness	27.4	4.2
Reflective Skepticism	15.7	2.4

The analysis revealed that EI demonstrated a strong positive correlation with critical thinking (*r* = 0.683, *p* < 0.001), indicating that higher EI is associated with stronger critical thinking abilities. Gender also demonstrated a significant correlation (*r* = 0.204, *p* < 0.001), suggesting differences in critical thinking skills based on gender. Additionally, having a family member in the medical or nursing field was positively correlated with critical thinking (*r* = 0.176, *p* = 0.003), suggesting that familial exposure to healthcare may be associated with enhanced critical thinking skills, as outlined in Table 3.

**Table 3:** Correlation between variables and critical thinking (*N* = 288)

Variable	Critical thinking (*r*, *p*-value)
Emotional intelligence	0.683 (< 0.001) **
Age	0.044 (0.459)
Gender (male)	0.204 (< 0.001) **
Academic year	0.007 (0.906)
Family member in healthcare (yes)	0.176 (0.003) **
Interest in nursing (yes)	0.098 (0.096)

** Correlation significant at *p* < 0.01

A multiple linear regression analysis was conducted to identify predictors of critical thinking among nursing students. The overall model was statistically significant (*p* < 0.001, R² = 0.491, adjusted R² = 0.486), indicating that 49.1% of the variance in critical thinking was explained by the predictors. Emotional intelligence was the strongest predictor, with a standardized beta coefficient (β = 0.658, *p* < 0.001), suggesting that higher emotional intelligence is strongly associated with enhanced critical thinking abilities. Gender also showed a significant influence (β = 0.131, *p* = 0.002), indicating that male nursing students demonstrated higher critical thinking skills compared to their female counterparts. Additionally, having a family member in the medical or nursing profession was a significant predictor (β = 0.087, *p* = 0.043), suggesting that students with familial exposure to healthcare settings may develop stronger critical thinking abilities, as shown in Table 4.

**Table 4:** Predictors of critical thinking (Multiple linear Regression)

Predictor	Unstandardized B	Standardized β	t	*p*-value	95% CI
Emotional intelligence	0.249	0.658	15.322	< 0.001	[0.217, 0.281]
Gender (male)	1.588	0.131	3.068	0.002	[0.569, 2.607]
Family member in healthcare (yes)	1.131	0.087	2.029	0.043	[0.034, 2.229]

## Discussion

This study examined the relationship between emotional intelligence (EI) and critical thinking (CT) among Palestinian nursing students who face political instability, resource deficiencies, and educational obstacles within a specific context. The findings revealed a strong significant correlation between EI and CT (*r* = 0.683), with EI emerging as the strongest predictor of CT. Students showed high EI scores (M = 122.5 ± 16.1), particularly in utilizing emotions, whereas CT disposition was in the moderate range overall (M = 43.1 ± 6.1). These results are consistent with international studies emphasizing the interplay between emotional and cognitive abilities in nursing practice. Similarly, Hasan and Noor identified a similar correlation between EI and CT (*r* = 0.71) among Saudi nursing students [12], suggesting that emotional regulation facilitates analytical reasoning in Arab cultures as well. Fereidouni et al. [13] also found that Iranian nursing students with higher EI demonstrated enhanced clinical judgment, supporting the universality of this relationship in resource-limited settings. However, the strength of this relationship differs from that found by Meyer in an American sample (*r* = 0.42), where more formal EI training and institutional support may reduce reliance on self-initiated emotional regulation. This contrast may reflect how structural stressors in conflict-affected contexts like Palestine potentially heighten the reliance on EI for addressing clinical challenges [14]. Recent empirical and review evidence suggests several plausible mechanisms explaining this association. First, EI facilitates the regulation of stress responses, which can preserve cognitive resources for reflective judgment during clinical problem-solving [4, 25]. Second, the ability to perceive and manage emotions can promote metacognitive monitoring, allowing students to evaluate and adjust their reasoning strategies in real time [26]. These mechanisms are particularly relevant in the Palestinian context, where frequent exposure to uncertainty and high-pressure healthcare environments may foster a greater integration of emotional regulation into clinical reasoning processes.

The moderate CT scores observed in this study warrant deeper analysis. Several factors may contribute to these findings, including the emphasis on traditional pedagogical approaches in Palestinian nursing education, which may prioritize knowledge acquisition over analytical skill development [15]. Additionally, the high-stress learning environment created by political instability and resource constraints may impact students’ ability to engage in reflective thinking processes essential for CT development. The moderate scores in both CT subscales, critical openness and reflective skepticism, suggest that while students demonstrate some analytical capabilities, there is substantial room for improvement through targeted educational interventions.

Interestingly, male students scored higher in CT, a finding that contrasts with global patterns in the female-majority nursing profession but aligns with regional studies. Cultural factors in Palestinian society may contribute to this finding, as men are often culturally expected to assume decision-making roles during stressful situations, which may foster CT development through early preparation for assertiveness and crisis-resolution [18]. For example, family and societal expectations regarding healthcare leadership roles, particularly important in a sector experiencing stress due to political uncertainty, may encourage male students to develop analytical skills early. In contrast, studies in Western contexts, such as Nieto [27], support findings of no gender difference in CT, reflecting egalitarian education standards. This contrast underscores the need for developing gender-sensitive pedagogies that consider cultural dynamics to address potential biases in educational environments.

The role of family healthcare exposure in predicting CT identified a statistical association. Elnouby et al. [28] in Egypt and Raghubir [29] in India observed that healthcare mentorship from relatives may shape clinical reasoning and ethical awareness, which could explain why family healthcare exposure, reported by 68.4% of the sample, may contribute to the early development of reflective skepticism and problem-solving. Potential pathways may include: (1) early exposure to clinical reasoning discussions during family conversations about healthcare cases, (2) informal mentoring where family members share problem-solving approaches used in clinical practice, (3) enhanced understanding of healthcare contexts that facilitates faster adaptation to clinical thinking patterns, and (4) increased motivation to develop analytical skills through role modeling. In Palestine, where clinical training opportunities may be limited due to overcrowded hospitals, family mentorship may provide alternative learning environments where students observe real-world application of clinical decision-making processes. However, the specific pathways through which family healthcare exposure influences CT development remain unclear and warrant future investigation using qualitative methods to understand these mentorship dynamics.

The moderate CT scores found in this study (M = 43.1 ± 6.1) are comparable to findings in neighboring countries. Jordanian nursing students achieved similar results on the CTDS (M = 44.2 ± 5.8), as reported by Malak et al. [30], while Turkish cohorts demonstrated higher levels of CT (M = 49.1 ± 4.9), as shown by Ghezzi et al. [31], possibly due to greater integration of active learning strategies. In contrast, Palestinian students’ high EI scores (M = 122.5) exceed those in Lebanon (M = 107.3), as found by Khademi et al. [3]. However, several limitations in the interpretation of these findings must be acknowledged. The attribution of high EI scores solely to ‘adaptive resilience in challenging environments’ extends beyond what the current data can support, as this study did not directly measure resilience or establish causal links between environmental stressors and EI development. Multiple factors could contribute to these higher scores, including cultural differences in emotional expression, variations in educational approaches, or demographic characteristics of the sample. Future research should incorporate direct measures of resilience and environmental stressors to test these proposed relationships more rigorously. Internationally, Finnish nursing students reported lower EI (M = 115.8), as noted by Talman et al. [32], suggesting that EI development may be enhanced in environments that require rapid emotional adaptation.

In the Palestinian nursing education system, emotional intelligence could be fostered through structured interventions such as simulation-based training, role-playing exercises, reflective journaling, and guided group discussions. These approaches would allow students to practice empathy, self-awareness, emotional regulation, and interpersonal skills in realistic clinical scenarios, thereby enhancing both EI and clinical thinking abilities.

However, several limitations in the interpretation of these findings must be acknowledged. The attribution of high EI scores solely to “adaptive resilience in unsafe environments” extends beyond what the current data can support, as this study did not directly measure resilience or establish causal links between environmental stressors and EI development. Future research should incorporate direct measures of resilience and environmental stressors to test these proposed relationships more rigorously.

### Strengths and limitations of the study

To the best of our knowledge, this is the first study in Palestine to investigate the relationship between emotional intelligence and clinical thinking among nursing students. This novelty strengthens the contribution of the present work by addressing a previously unexplored gap in the local context and providing baseline evidence that can guide future research, educational interventions, and policy development. Despite the valuable contributions of this study, several limitations must be considered. First, the cross-sectional design limits the ability to establish causal relationships between emotional intelligence and clinical thinking; future longitudinal or experimental studies are needed to confirm the directionality of these associations. Second, the study relied on self-report instruments, which may be subject to social desirability bias and inaccuracies in participants’ self-assessment. Third, the sample was drawn from a single institution, which may limit the generalizability of the findings to nursing students in other Palestinian universities or different educational contexts. Finally, the analysis did not account for potential unmeasured confounders, such as academic performance, prior clinical exposure, or personal life experiences, which could influence both emotional intelligence and clinical thinking.

### Recommendations

For nursing education policy-makers in Palestine, it is advisable to incorporate structured emotional intelligence development programs, such as simulation-based scenarios, role-play activities, and reflective practice sessions into nursing curricula. Also, future studies should use longitudinal designs to examine causal relationships between EI and CT, gender differences in CT, and employ mixed-methods designs to explore the nuances of cultural influences.

## Conclusion

This study established a strong relationship between emotional intelligence (EI) and critical thinking (CT) disposition among Palestinian nursing students, identifying EI as a significant predictor of CT in a context characterized by political conflict and resource constraints. The strong correlation demonstrates the interconnected nature of emotional and cognitive competencies in developing clinical competence, particularly in situations where systemic stressors may demand adaptive resilience. Cultural factors, such as gender roles and family healthcare exposure, also appear to shape this relationship, suggesting the need for context-specific educational strategies.

To address these challenges, the integration of EI training into nursing education, utilizing simulation-based teaching, reflective practice, and culturally-appropriate family-engagement mentorship, may enhance CT disposition while remaining culturally compatible with Palestinian cultural norms. These interventions should be supported by national reforms to shift from rote learning towards pedagogies emphasizing analytical thinking and emotional adaptability.

## Data Availability

No datasets were generated or analysed during the current study.
